# Predictors of psychiatric readmission within 12 months following discharge from inpatient units in Alberta, Canada

**DOI:** 10.1017/gmh.2026.10229

**Published:** 2026-05-22

**Authors:** Hossam Elgendy, Reham Shalaby, Ernest Owusu, Wanying Mao, Belinda Agyapong, Wes Vuong, Ejemai Eboreime, Nnamdi Nkire, Yifeng Wei, Vincent Agyapong

**Affiliations:** 1 Psychiatry, University of Alberta Faculty of Medicine & Dentistry, Canada; 2 Alberta Health Services, Canada; 3 Psychiatry, Dalhousie University, Canada

**Keywords:** admitted, inpatient, satisfaction, mental health, readmission, psychiatric units

## Abstract

Psychiatric readmissions remain a significant challenge for mental health systems and may reflect gaps in continuity of care and support. This study examined predictors of psychiatric readmission within 12 months of discharge from an acute inpatient admission, with particular attention to patient satisfaction with care. Data from 1,070 psychiatric service users were analyzed using multivariate logistic regression. Variables included sociodemographic and clinical characteristics, prior inpatient admission, and satisfaction with care received during the indexed admission. Prior inpatient admission within the previous 12 months was the strongest predictor of readmission (OR = 2.00, 95% CI [1.45–2.76], p < .001). Older age (>40 years) and higher educational attainment were associated with lower readmission risk, whereas younger age and lower educational attainment were associated with increased risk. Employment and housing status were also significant predictors. In contrast, patient satisfaction with inpatient care was not associated with readmission, nor were gender, ethnicity, relationship status, resilience, well-being, depression, anxiety, or study cluster. These findings suggest that psychiatric readmission is driven primarily by recent hospitalization history and sociodemographic factors rather than patient satisfaction, highlighting the importance of targeted discharge planning and psychosocial support to reduce future hospitalizations.

## Impact statement

Psychiatric readmissions place a substantial burden on individuals, families and mental health services, often disrupting recovery, straining social supports and increasing pressure on already limited inpatient resources. This study clarifies the key factors that most strongly influence the likelihood of readmission following discharge from psychiatric care, offering important guidance on where prevention efforts may be most effective. The broader impact of this research lies in its implications for service planning and policy, suggesting that mental health systems may benefit from prioritizing targeted discharge planning, structured follow-up and enhanced community-based supports for individuals with recent admissions and greater social vulnerability. By focusing resources on continuity of care, as well as education, employment support and housing stability, services may reduce avoidable readmissions, improve long-term outcomes and contribute to more sustainable and equitable mental health care systems.

## Introduction

Psychiatric readmission defined as re-hospitalization following discharge from inpatient psychiatric care is widely recognized as a key indicator of mental health service quality, continuity and effectiveness(Durbin et al., 2007; Vigod et al., 2013; Donisi et al., 2016). Frequent readmissions are costly, distressing for patients and their families and often signal inadequacies in treatment, discharge planning or postdischarge support(Byrne et al., 2010; Kalseth et al., 2016; Owusu et al., 2022). Globally, healthcare systems monitor early readmissions (within 30 to 90 days) as markers of inadequate care (Kansagara et al., 2011; Del Favero et al., 2020; Morel et al., 2020; Muhammad et al., 2023). For example, data from a study done in Finland indicated that 11% of psychiatric inpatients were readmitted within 30 days, and 33% within one year of discharge (Virtanen et al., 2024). National data from the Canadian Institute for Health Information (CIHI) showed that 30-day readmission rates for mental-health and substance-use conditions vary considerably across provinces and territories, ranging from approximately 10% to over 15%, with some northern regions reporting even higher rates (“https://www.cihi.ca/en/indicators/30-day-readmission-for-mental-health-and-substance-use”). Studies from individual provinces, including Ontario and Alberta, similarly report 30-day readmission rates generally between 9% and 10%, while certain high-risk groups, such as individuals experiencing homelessness face substantially elevated rates (Barker et al., 2018; Slomp et al., 2012; Alberta Health Services, 2023). These interprovincial differences highlight the influence of discharge planning, availability of community supports and broader social determinants of health on readmission patterns. Some studies have identified a range of clinical and sociodemographic determinants that predict psychiatric readmission, particularly, clinical characteristics (especially psychotic and mood disorders), prior hospitalizations, unemployment, unstable housing and limited social support (Donisi et al., 2016; Sfetcu et al., 2017; Pasqualini et al., 2024; Virtanen et al., 2024). Organizational factors such as staff-to-patient ratios, ward environment and care coordination have also been implicated (Sfetcu et al., 2017). Although increasing attention is being directed toward patient-reported experiences of care, including satisfaction with inpatient services, as potentially modifiable contributors to postdischarge outcomes, their integration with established predictors in multivariate models remains limited (Donisi et al., 2016; Yamaguchi et al., 2025). We often assess satisfaction aspects using patient-reported measures, feedback surveys or satisfaction questionnaires filled out during or after hospitalization (Birkelien, 2017; Haji et al., 2022). Theoretically, positive inpatient satisfaction may be associated with improved postdischarge outcomes, including reduced readmission risk. Proposed mechanisms include, enhanced therapeutic alliance, better medication adherence, better understanding of discharge plans and improved coping with posthospitalization stressors (Zendjidjian et al., 2014; Donisi et al., 2016; Yamaguchi et al., 2025). Despite these theoretical pathways, findings in the literature remain mixed. Some studies report significant associations between satisfaction with inpatient care and readmission, while others find no clear link likely due to variations in measurement tools, timeframes, study design and the multifactorial nature of the readmission process (Donisi et al., 2016; Shields et al., 2023; Yamaguchi et al., 2025). Interestingly, most existing research focuses on Satisfaction as a predictor of short-term readmission (within 30 days), potentially overlooking its effect on long-term outcomes (Donisi et al., 2016; Chen et al., 2018; Muhammad et al., 2023; Ren et al., 2024). In the context of psychiatric readmission, patient satisfaction should be understood as one component within a broader set of clinical and sociodemographic determinants rather than a standalone predictor. Despite extensive research on psychiatric readmission, several important gaps remain. Much of the existing literature has focused on short-term outcomes, with limited evidence on longer-term readmission risk beyond 30 or 90 days. In addition, patient-reported experience measures, including satisfaction with inpatient care, have not been consistently examined alongside established clinical and sociodemographic predictors within unified multivariate models (Donisi et al., 2016). Furthermore, there is a lack of real-world, multicenter studies within the Canadian context that reflect routine clinical practice. Addressing these gaps, the present study aims to examine the predictors of psychiatric readmission within 12 months following discharge from inpatient psychiatric units in Alberta, Canada, to identify the characteristics most strongly associated with long-term readmission risk, while also evaluating the role of patient satisfaction as a secondary factor within a comprehensive predictive framework. By doing so, this study seeks to inform targeted interventions that enhance patient care and reduce avoidable hospital readmissions.

## Methodology

### Study design and data collection

This study was conducted in Alberta, Canada. As of July 1, 2023, the provincial population was estimated at 4,695,290 residents, according to the Government of Alberta. Data for this paper include patients recruited between March 8, 2022, and February 10, 2024. Participants were recruited across all the acute mental health units in three main cities: Edmonton, Calgary, and Grande Prairie, strategically selected for their geographic diversity and participation in a broader regional initiative. The study employed a pragmatic stepped-wedge cluster-randomized design to recruit participants, where the primary goal of the study was to evaluate the effects of sociodemographic, clinical characteristics and supportive text messages (Text4Support) and peer support services (PSS) on individuals with mental illness following their discharge from acute mental health facilities in Alberta (Eboreime et al., 2022). A substudy was conducted at the same setting to assess inpatient satisfaction with hospital care. Recruitment was supported by unit managers, physicians and nurses at each site, who helped identify eligible participants. Following informed consent via a paper-based form, participants completed two self-administered online surveys using Redcap (Research Electronic Data Capture), a secure web-based platform designed for survey management (Harris et al., 2019). The survey instruments (attached as a Supplementary Files) were developed by the Decision Support Unit of the Edmonton Zone, Alberta Health Services (Addiction and Mental Health Program) (https://www.albertahealthservices.ca/amh/amh.aspx.). The surveys were administered during the participants’ inpatient stay (within the week prior to discharge) and took approximately 15–20 min to complete. Anonymity and confidentiality of responses were emphasized. The first survey collected sociodemographic information, including age, sex, ethnicity, marital status, housing status and education. Psychiatric diagnoses were initially self-reported by participants and subsequently verified through clinical records at the time of recruitment. The second survey was developed to assess patients’ satisfaction with psychiatric inpatient hospital care. The survey included both quantitative and qualitative questions. For the purpose of this study, only the quantitative questions were applied. Responses were recorded using a 5-point Likert scale, later consolidated into three categories for analysis: “Yes” (including “Yes, definitely” and “Yes, to some extent”), “Neutral” and “No” (including “Not really” and “Definitely not”). Development of the survey instrument was guided by expert consultation, including clinicians, mental health practitioners and health services researchers, to ensure content validity, relevance and clarity. The final version was compared against established validated instruments like *Hospital Consumer Assessment of Healthcare Providers and Systems* (HCAHPS), and *Picker Patient Experience Questionnaire (*PPE-15) to further support its validity (Wolf et al., 2012; Indovina et al., 2021). Instrument reliability was evaluated using Cronbach’s alpha, yielding a value of 0.70 in a sample of 1,070 participants, indicating acceptable internal consistency (Tavakol and Dennick, 2011; García-Batista et al., 2018). To mitigate response biases such as social desirability, participation was emphasized as voluntary and anonymous, and standardized Likert response scales were employed to reduce external influence on participants’ answers. Participants’ readmission status within 12 months postdischarg**e** was obtained through Linkage to administrative health records, subject to data-sharing agreements and ethics approval. Psychiatric diagnoses were categorized into major diagnostic groups (e.g., mood disorders, anxiety disorders, psychotic disorders, and substance use disorders), and clinical measures were assessed at discharge using the Patient Health Questionnaire-9 (PHQ-9), the Generalized Anxiety Disorder-7 (GAD-7), the Brief Resilience Scale (BRS), and the World Health Organization Well-Being Index (WHO-5), respectively.

### Ethics statement

The University of Alberta’s Health Research Ethics Board approved this study (Ref # Pro00111459), and the regional health authority granted further operational approval. Before being included in the study, individuals signed written informed consent forms.

### Inclusion and exclusion criteria

The study selection criteria for participants adhered to those outlined in the published study protocol for the primary study, which aimed to assess the impact of supportive text messages (Text for Support) and peer support services (PSS) on individuals with mental illness following their discharge from acute mental health facilities in Alberta (Eboreime et al., 2022). In summary, participants needed to be diagnosed with a mental illness, be at least eighteen years old, scheduled for discharge from an inpatient psychiatric facility and possess a mobile phone device with an active phone number. Additionally, participants had to be capable of receiving and reading English text messages and have the capacity to provide informed consent.

### Outcome measures

The primary outcome of this study was psychiatric readmission within 12 months postdischarge. Predictor variables included sociodemographic, clinical diagnosis, study clusters, including treatment as usual (TAU), Supportive Text Messages (SMS / Text4Support) and Supportive Text Messages plus Peer Support (SMS + PSS) and service-related factors, including patient satisfaction with inpatient care.

### Statistical analysis

Data analysis was conducted using SPSS version 25 for Mac (IBM Corp., USA) (IBM Support, 2017). Descriptive statistics were used to examine the sociodemographic and clinical characteristics of participants in relation to psychiatric admission within the preceding 12 months. Multiple binary logistic regression model was applied to identify key predictors of hospital readmission within 12 months postdischarge (Menditto et al., 2006). The model included the variables of prior inpatient admission, satisfaction with inpatient care, age, gender, ethnicity, education, employment, housing, relationship status and several mental health screening measures (PHQ-9, GAD-7, WHO-5 and the Brief Resilience Scale). Odds ratios (ORs) and corresponding confidence intervals were calculated to assess the strength and significance of these predictor variables.

## Results

Table 1 represents the distribution of participants’ demographic and clinical characteristics stratified by whether they had experienced a prior psychiatric inpatient admission in the 12 months preceding their current hospital admission. A total of 1,070 participants were included in the study. The largest share of participants were aged 25 years or younger, followed by those aged 26–40 years and those older than 40. Slightly more than half were female, and the majority reported Caucasian ethnicity, with Asian, Black, Indigenous, or mixed/other groups being represented in smaller numbers. Educational levels varied, although over half had completed high school, and about two in five had postsecondary education. Most participants were single, while fewer were married or in common-law relationships. Employment status showed that over half were unemployed, while the remainder were employed, students, retired or belonged to other categories. In terms of housing, the largest proportion lived with family or friends, followed by those renting or owning their home. More than half of the participants reported being satisfied or very satisfied with inpatient services. Anxiety and mood disorders were the most common diagnoses, followed by substance use, personality, or other disorders and then psychotic disorders. On the Brief Resilience Scale, a little under half showed normal to high resilience, while the remainder demonstrated low resilience. Well-being scores on the WHO-5 were almost evenly split between good and poor well-being. Based on screening tools, just over half had depression, while about one in three had anxiety. Within 12 months following discharge, about one-third of the participants experienced psychiatric readmission.

**Table 1.** Distribution of demographic and clinical characteristics of the participants against 12-month prior admission variable Table 1. long description.

Variables	No prior inpatient admissions *n* = 385	Prior inpatient admissions *n* = 685	Total*n* = 1,070
**Age**			
≤ 25 y	94 (23.8)	301 (76.2)	395 (36.9)
26–40y	151 (41.3)	214 (58.6)	365 (34.1)
>40y	140 (45.2)	170 (54.8)	310 (29.0)
**Gender**			
Males	149 (33)	303 (67)	452 (42.2)
Females	222 (37.9)	364 (62.1)	586 (54.8)
other	14 (43.8)	18 (56.3)	32 (3.0)
**Ethnicity**			
Caucasian	240 (36.3)	421 (63.7)	661 (61.8)
Indigenous	42 (42.9)	56 (57.1)	98 (9.2)
Black	37 (31.9)	79 (68.1)	116 (10.8)
Asian	38 (31.4)	83 (68.6)	121 (11.3)
Mixed/Other	28 (37.8)	46 (62.2)	74 (6.9)
**Education level**			
Less than High School	14 (35.9)	25 (64.1)	39 (3.6)
High School Diploma	177 (31.9)	377 (68.1)	554 (51.8)
Post-secondary Education	183 (41.3)	260 (58.7)	443 (41.4)
Prefer not to say	11 (32.4)	23 (67.6)	34 (3.2)
**Relationship status**			
Married/Partnered/Common-Law	134 (43.1)	177 (56.9)	311 (29.1)
Single	205 (32.4)	428 (67.6)	633 (59.2)
Separated or Divorced	35 (42.2)	48 (57.8)	83 (7.8)
Widowed	5 (41.7)	7 (58.3)	12 (1.1)
Prefer not to say	6 (19.4)	25 (80.6)	31 (2.9)
**Employment status**			
Employed	137 (43.2)	180 (56.7)	317 (29.6)
Unemployed	180 (31.7)	388 (68.3)	568 (53.1)
Student	29 (33.3)	58 (66.7)	87 (8.1)
Retired	29 (46)	34 (54)	63 (5.9)
Other	10 (28.6)	25 (71.4)	35 (3.3)
**Housing status**			
Own Home	102 (48.3)	109 (51.7)	211 (19.7)
Rented Accommodation	131 (38)	214 (47.9)	345 (32.3)
Live with Family or Friends	133 (29.8)	314 (70.2)	447 (41.8)
Other (Couch Surfing, Shelter, Street)	18 (27.3)	48 (72.7)	66 (6.2)
**Level of satisfaction with the care received during the indexed admission**			
Very satisfied (9–10)	134 (39.4)	206 (60.6)	340 (36.4)
Satisfied (6–8)	163 (33.2)	328 (66.8)	491 (52.5)
Not Satisfied (<6)	34 (32.7)	70 (67.3)	104 ((11.1)
**Mental health diagnosis**			
Anxiety/Mood	242 (37.9)	397 (62.1)	639 (59.8)
Psychosis	53 (30.6)	120 (69.4)	173 (16.2)
SUD/Personality disorder/Other	89 (34.6)	168 (65.4)	257 (24.0)
**Brief Resilience Scale Category (BRS)**			
Normal to High Resilience	154 (33)	312 (67)	466 (43.6)
Low Resilience	230 (38.1)	373 (61.9)	603 (56.4)
**WHO-5 Category**			
Good Wellbeing	178 (32.5)	370 (67.5)	548 (51.3)
Poor Wellbeing	206 (39.6)	314 (60.4)	520 (48.7)
**Depression Category (PHQ-9)**			
Unlikely Depression	166 (36.3)	291 (63.7)	457 (42.8)
Likely Depression	218 (56.8)	393 (57.5)	611 (57.2)
**Anxiety Category (GAD-7)**			
Unlikely anxiety	222 (32.9)	453 (67.1)	675 (63.6)
Likely anxiety	159 (41.7)	228 (58.9)	387 (36.4)
**Readmission within 12 months (post-index Admission)**			
No	304 (41.1)	435 (58.9)	739 (67.1)
Yes	89 (24.6)	273 (75.4)	362 (32.9)
**Cluster**			
SMS	112 (36.2)	197 (63.8)	309 (28.1)
SMS + PS	111 (30.8)	249 (69.2)	360 (32.7)
TAU	170 (39.3)	262 (60.6)	432 (39.2)

Table 2 illustrates the results of a binary logistic regression analysis predicting the likelihood of readmission within 12 months following the index admission. Fifteen independent variables were entered into the model, including age category, gender, ethnicity, education, current relationship status, employment status, current housing status, inpatient satisfaction, mental health diagnosis, Brief Resilience Scale (BRS) category, WHO-5 wellbeing category, PHQ-9 depression category, GAD-7 anxiety category, study cluster and admission status 12 months prior to the index admission. The overall regression model was statistically significant, Χ^2^ (31, *N* = 929) = 81.69, *p* = <.001, indicating that the predictors, taken together, reliably distinguished between participants who were readmitted and those who were not. The model explained between 8.4% (Cox & Snell R^2^) and 11.7% (Nagelkerke R^2^) of the variance in readmission status and correctly classified 76.0% of cases. The model revealed that prior admission status, age, education level, employment status and housing status were significant predictors of readmission. Participants with a prior admission were approximately twice as likely to be readmitted (*B* = 0.694, *p* < 0.001, OR = 2.00, 95% CI [1.45, 2.78]) compared to those without a previous hospitalization. Participants aged over 40 years were significantly less likely to be readmitted compared to those 25 years or younger (*B* = −0.532, *p* = 0.034, OR = 0.59, 95% CI [0.36, 0.96]). Educational achievement also revealed a protective effect, with respondents holding a high school diploma (*B* = −0.861, *p* = 0.022, OR = 0.42, 95% CI [0.20, 0.88]) or postsecondary education (*B* = −0.940, *p* = 0.014, OR = 0.39, 95% CI [0.18, 0.83]) being significantly less likely to experience readmission compared to those with less than a high school education. Employment status was also significant (χ^2^ (4) = 9.654, *p* = 0.047), with unemployed individuals showing increased odds of readmission (*B* = 0.419, *p* = 0.025, OR = 1.52, 95% CI [1.05, 2.20]). Housing status was significant (χ^2^ (3) = 8.881, *p* = 0.031), suggesting that participants with unstable housing (e.g., couch surfing, shelters, or homelessness) possibly more likely to be readmitted (*B* = 0.538, OR = 1.71, 95% CI [0.85, 3.46]). In contrast, study cluster, inpatient satisfaction, gender, ethnicity, relationship status and clinical and well-being measures (resilience, wellbeing, depression and anxiety) were not significantly associated with readmission (*p* > 0.05). Overall, within 12 months postdischarge, approximately one-third of participants (33%) experienced psychiatric readmission.

**Table 2.** Multivariate logistic regression model for respondents that predicts the likely readmission within 12-months post-index admission among participants Table 2. long description.

Characteristics	B	SE	Wald	df	*p* value	OR	95% C.I.	
							Upper	Lower
**Study cluster**								
SMS			1.213	2	.545			
SMS + PS	−.144	.199	.527	1	.468	.866	.586	1.278
TAU	.059	.179	.108	1	.742	1.061	.746	1.508
**Inpatient satisfaction category**								
Very Satisfied			2.291	2	.318			
Satisfied	.231	.163	2.006	1	.157	1.259	.915	1.733
Not Satisfied	.266	.252	1.115	1	.291	1.305	.769	2.141
**Age categories (years)**								
≤ 25 years			7.149	2	.028			
26–40 years	.041	.195	.043	1	.835	1.041	.711	1.525
>40 years	−.532	.252	4.470	1	.034	.587	.358	.962
**Gender**								
Male			2.030	2	.362			
Female	.049	.156	.099	1	.753	1.050	.774	1.425
Other	−.631	.485	1.689	1	.194	.532	.206	1.378
**Ethnicity category**								
Caucasians			6.037	4	.196			
Indigenous People	.008	.259	.001	1	.974	1.008	.607	1.675
Black People	−.394	.257	2.341	1	.126	.675	.407	1.117
Asians	.044	.248	.031	1	.860	1.045	.642	1.700
Other	−.651	.332	3.848	1	.050	.521	.272	.999
**Education category**								
Less than High School			6.340	3	.096			
High School Diploma	−.861	.375	5.279	1	.022	.423	.203	.881
Post-Secondary Education	−.940	.384	6.006	1	.014	.390	.184	.828
Other	−.632	.540	1.368	1	.242	.532	.184	1.532
**Current relationship status**								
Partnered/Married/Common law.			4.592	4	.332			
Single	.037	.187	.039	1	.844	1.037	.719	1.497
Separated /Divorced	−.151	.324	.216	1	.642	.860	.456	1.623
Widowed	−.951	.847	1.260	1	.262	.386	.073	2.033
Other	.858	.506	2.878	1	.090	2.359	.875	6.359
**Current employment status**								
Employed			9.654	4	.047			
Unemployed	.419	187	5.016	1	.025	1.520	1.054	2.193
Student	−.307	.341	.811	1	.368	.736	.377	1.435
Retired	.573	.380	2.277	1	.131	1.774	.843	3.733
Other	.202	.471	.185	1	.667	1.224	.486	3.082
**Current housing status**								
Own Home			8.881	3	.031			
Rented Accommodation	.088	.233	.144	1	.704	1.092	.692	1.724
Live with Family/Friends	−.312	.255	1.494	1	.222	.732	.444	1.207
Couch/Shelter/Street/Other	.538	.357	2.269	1	.132	1.712	.850	3.448
@12Monthpriorinpatient Admitted	.694	.166	17.596	1	.000	2.002	1.448	2.770
**BRS category**								
Normal to High Resilience								
Low Resilience	−258	.165	2.452	1	.117	.773	.559	1.067
**WHO category**								
Good Well-being								
Poor Well-being	.215	.176	1.494	1	.222	1.240	.878	1.751
**Depression Cat. (PHQ)**								
Likely Depression								
Unlikely Depression	.090	.183	.241	1	.624	1.094	.764	1.567
**Anxiety Cat. (GAD)**								
Likely Anxiety								
Unlikely Anxiety	−286	.184	2.409	1	.121	.751	.523	1.078
Constant	−.396	.515	.591	1	.442	.673		

## Discussion

The study aimed to identify the predictors of psychiatric readmission within 12 months postindex admission. The analysis of participant characteristics revealed various significant determinants to psychiatric patients’ readmission. Readmission rate within 12 months postindex admission was almost twice higher among those with prior admission (38.6%) compared to those without (22.6%), underscoring the strong predictive value of recent inpatient care. This finding is supported by a number of literature sources emphasizing the importance of the “revolving door” phenomenon in psychiatric care, where individuals with recent admissions are at higher risk for recurrence and readmission (Lin et al., 2010; Di Giovanni et al., 2020; Lassemo et al., 2021). The finding also underlines the cyclical nature of psychiatric hospitalization and highlights the need for intensive postdischarge planning and follow-up for individuals with recent inpatient histories (Durbin et al., 2007; Steffen et al., 2009; Stefan et al., 2013; Tulloch et al., 2016). Sociodemographic variables also played a significant role in predicting readmission risk. Age came as a significant factor wherein younger individuals were more likely to have readmission within 12 months post discharge. This finding is consistent with previous studies (Xueyan Han et al., W David Lohr et al., and Lin Chen et al.), which reported that youth 25 years and less have higher readmission rates than the older age category (Lin et al., 2010; Han et al., 2020; Lohr et al., 2024). This may reflect developmental vulnerabilities and lower levels of treatment engagement among younger adults (Anderson and Steinberg, 1985; Silverstein et al., 2008). Level of education was also significantly associated with readmission, particularly among individuals with lower educational attainment. Higher education is associated with better health outcomes; our findings suggest that individuals in these education categories may possess practical coping or problem-solving skills that reduce the likelihood of psychiatric crisis or may be more engaged with community-based services. This finding aligned with the studies done by Kalseth et al. and Elhassan et al. who reported that communities with higher education had a lower likelihood of psychiatric readmission (Kalseth et al., 2016; Elhassan et al., 2020). Relationship status also differed significantly. Being single was more common among those with a recent admission history, while those in partnered or married relationships were more represented in the no-prior group. Social support and relational stability are known protective factors against psychiatric relapses and may buffer against the need for hospitalization according to Corrigan and Phelan (2004). Employment status was another important factor, with unemployment being significantly associated with increased readmission risk. This finding is consistent with previous literature indicating that unemployment is a risk factor for both poor mental health outcomes and recurrent hospital admissions (Sareen et al., 2005; Sledge and Dunn, 2008; Schmutte et al., 2009; Compton and Shim, 2015; Russolillo et al., 2023). Employment instability may predispose to stress, less access to resources and interrupted care continuity, consequently increasing the likelihood of hospitalization. Similarly, housing status emerged as a significant overall predictor. Although no individual category reached significance, the overall association supports existing evidence linking housing instability to increased psychiatric service utilization (Fazel et al., 2008; Kerman et al., 2018; Laliberté et al., 2019). Unstable housing environments may exacerbate symptoms, limit access to treatment or disrupt medication adherence. Contrary to expectations, patient satisfaction was not a significant predictor for psychiatric readmission within 12 months postdischarge in this study. Our findings suggest that satisfaction alone may not be a sufficient predictor of long-term clinical outcomes such as readmission. This may reflect the complex and multifactorial nature of readmission, where structural, socioeconomic and clinical factors outweigh subjective perceptions of care quality. Additionally, the patient satisfaction measure used in this study, while demonstrating acceptable reliability, did not allow for detailed analysis of specific satisfaction domains, which may have limited the ability to detect associations with readmission outcomes. This finding aligned with the results of other studies, which found *no* significant association between satisfaction with inpatient care and later readmission during follow-up (Lyons et al., 1997; Lien, 2002; Donisi et al., 2016; Yamaguchi et al., 2025). Psychiatric disorders screening measures, including depression (PHQ-9), anxiety (GAD-7), Brief Resilience Scale (BRS), and overall well-being (WHO-5), did not significantly predict readmission in this model. Although these variables are clinically meaningful and often associated with psychiatric outcomes, their lack of statistical significance in this model may be due to overlapping with other factors such as sociodemographic and prior admission. These results are supported by existing literature (Lyons et al., 1997; Vijayaraghavan et al., 2015; Lee et al., 2021). In this study, assignment to intervention groups (treatment as usual, supportive text messaging, and supportive text messaging combined with peer support) was not significantly associated with psychiatric readmission within 12 months postdischarge. This finding suggests that, within the context of this analysis, these postdischarge supportive interventions did not independently influence long-term readmission risk. One possible explanation is that the effects of such interventions may be more evident in the short term rather than over extended follow-up periods. In addition, the impact of these interventions may be moderated by broader structural and sociodemographic factors, such as housing stability, employment status, and prior hospitalization history, which were identified as significant predictors in this study. Furthermore, variability in participant engagement with the interventions may have influenced their effectiveness (Steffen et al., 2009; Donisi et al., 2016; Obuobi-Donkor et al., 2025). Taken together, these findings highlight the complexity of reducing psychiatric readmissions and suggest that while supportive interventions may contribute to patient well-being, they may need to be integrated with more comprehensive, system-level strategies to meaningfully reduce long-term readmission rates. Inpatient hospital satisfaction, postdischarge supportive interventions and psychiatric diagnosis are key to care quality and clinical assessment. However, these tools may be more effective in encouraging patient engagement and treatment adherence than in predicting readmission outcomes. Future research should focus on investigating the relationships between sociodemographic factors, clinical factors and psychiatric readmission. This approach will help identify populations at risk more accurately and understand the complex interactions that influence readmission. Further examination of patient satisfaction during hospitalization using more detailed satisfaction measures could reveal important effects on readmission risk. Additionally, expanding research to different healthcare settings and populations would improve generalizability. Clinically, the study findings reveal the need for prioritizing follow-up care for people with recent hospitalization, especially for younger patients and those with prior admissions and lower education levels, as they seem to face a higher risk of readmission. Providing employment support and housing stability may also decrease the chances of readmission. Mental health services should develop programs to build resilience and create smart discharge plans to tackle factors linked to readmission. Overall, improving continuity of care and addressing social factors that affect health may significantly improve patient outcomes and reduce the problem of repeated psychiatric hospitalizations.

## Limitations

Several limitations should be noted when looking at the findings of this study. Some factors, including patient satisfaction and clinical characteristics, depended on self-reported data. These data may be affected by recall bias or social desirability bias, which could influence the accuracy of responses. Additionally, the patient satisfaction measure used in this study, while demonstrating acceptable reliability, did not allow for detailed analysis of specific satisfaction domains, which may have limited the ability to detect associations with readmission outcomes. Also, the study sample might not fully represent the wider population of psychiatric patients, especially those who are not engaged with services or harder to reach. Furthermore, diagnostic categories were grouped broadly, which may mask important differences between psychiatric disorders and their individual effects on the risk of readmission. Important factors like medication adherence and the quality of social support were not included. Additionally, as the study took place within one healthcare system and geographic area, the findings might not apply to other settings with different patient populations, resources or care models which limits generalizability. Despite these limitations, the study offers important insights into the social, demographic and clinical factors linked to psychiatric readmission. It also points out areas for future research and targeted intervention.

## Conclusion

This study outlines important social and clinical factors linked to psychiatric readmission within 12 months postdischarge. Prior admission within twelve months was the strongest predictor. Younger age, employment status and lower education levels also had a significant impact on the readmission risk. Patient satisfaction with inpatient care, clinical diagnosis and postdischarge supportive interventions were not significantly linked to readmission, suggesting that while these factors are still important for overall care quality, it may not directly affect readmission outcomes. These findings highlight how sociodemographic factors and prior hospitalizations shape the risk of readmission. Ongoing studies are necessary to improve predictive models and create effective interventions for vulnerable groups.

## Supporting information

10.1017/gmh.2026.10229.sm001 Elgendy et al. supplementary material Elgendy et al. supplementary material

## Data Availability

The data presented in this study are available on request from the corresponding author. The data are not publicly available due to privacy and ethical reasons.

## Supplementary material

The supplementary material for this article can be found at http://doi.org/10.1017/gmh.2026.10229.

## Long descriptions

### Table 1. Long description

The table presents variables grouped as age, gender, ethnicity, education level, relationship status, employment status, housing status, satisfaction with care, mental health diagnosis, Brief Resilience Scale, W H O dash 5, depression, anxiety, readmission, and cluster. For each, categories are listed vertically. Columns are: no prior inpatient admissions (n equals 385), prior inpatient admissions (n equals 685), and total (n equals 1,070).

Age: 94 participants aged 25 years or younger had no prior admission (23.8 percent), 301 had prior admission (76.2 percent), total 395 (36.9 percent). For ages 26 to 40: 151 no prior (41.3 percent), 214 prior (58.6 percent), total 365 (34.1 percent). Over 40: 140 no prior (45.2 percent), 170 prior (54.8 percent), total 310 (29.0 percent).

Gender: Males 149 no prior (33 percent), 303 prior (67 percent), total 452 (42.2 percent). Females 222 no prior (37.9 percent), 364 prior (62.1 percent), total 586 (54.8 percent). Other 14 no prior (43.8 percent), 18 prior (56.3 percent), total 32 (3.0 percent).

Ethnicity: Caucasian 240 no prior (36.3 percent), 421 prior (63.7 percent), total 661 (61.8 percent). Indigenous 42 no prior (42.9 percent), 56 prior (57.1 percent), total 98 (9.2 percent). Black 37 no prior (31.9 percent), 79 prior (68.1 percent), total 116 (10.8 percent). Asian 38 no prior (31.4 percent), 83 prior (68.6 percent), total 121 (11.3 percent). Mixed or other 28 no prior (37.8 percent), 46 prior (62.2 percent), total 74 (6.9 percent).

Education level: Less than high school 14 no prior (35.9 percent), 25 prior (64.1 percent), total 39 (3.6 percent). High school diploma 177 no prior (31.9 percent), 377 prior (68.1 percent), total 554 (51.8 percent). Post-secondary 183 no prior (41.3 percent), 260 prior (58.7 percent), total 443 (41.4 percent). Prefer not to say 11 no prior (32.4 percent), 23 prior (67.6 percent), total 34 (3.2 percent).

Relationship status: Married or partnered 134 no prior (43.1 percent), 177 prior (56.9 percent), total 311 (29.1 percent). Single 205 no prior (32.4 percent), 428 prior (67.6 percent), total 633 (59.2 percent). Separated or divorced 35 no prior (42.2 percent), 48 prior (57.8 percent), total 83 (7.8 percent). Widowed 5 no prior (41.7 percent), 7 prior (58.3 percent), total 12 (1.1 percent). Prefer not to say 6 no prior (19.4 percent), 25 prior (80.6 percent), total 31 (2.9 percent).

Employment status: Employed 137 no prior (43.2 percent), 180 prior (56.7 percent), total 317 (29.6 percent). Unemployed 180 no prior (31.7 percent), 388 prior (68.3 percent), total 568 (53.1 percent). Student 29 no prior (33.3 percent), 58 prior (66.7 percent), total 87 (8.1 percent). Retired 29 no prior (46 percent), 34 prior (54 percent), total 63 (5.9 percent). Other 10 no prior (28.6 percent), 25 prior (71.4 percent), total 35 (3.3 percent).

Housing status: Own home 102 no prior (48.3 percent), 109 prior (51.7 percent), total 211 (19.7 percent). Rented 131 no prior (38 percent), 214 prior (62 percent), total 345 (32.3 percent). Live with family or friends 133 no prior (29.8 percent), 314 prior (70.2 percent), total 447 (41.8 percent). Other (couch surfing, shelter, street) 18 no prior (27.3 percent), 48 prior (72.7 percent), total 66 (6.2 percent).

Satisfaction with care: Very satisfied 134 no prior (39.4 percent), 206 prior (60.6 percent), total 340 (36.4 percent). Satisfied 163 no prior (33.2 percent), 328 prior (66.8 percent), total 491 (52.5 percent). Not satisfied 34 no prior (32.7 percent), 70 prior (67.3 percent), total 104 (11.1 percent).

Mental health diagnosis: Anxiety or mood 242 no prior (37.9 percent), 397 prior (62.1 percent), total 639 (59.8 percent). Psychosis 53 no prior (30.6 percent), 120 prior (69.4 percent), total 173 (16.2 percent). S U D or personality disorder or other 89 no prior (34.6 percent), 168 prior (65.4 percent), total 257 (24.0 percent).

Brief Resilience Scale: Normal to high resilience 154 no prior (33 percent), 312 prior (67 percent), total 466 (43.6 percent). Low resilience 230 no prior (38.1 percent), 373 prior (61.9 percent), total 603 (56.4 percent).

W H O dash 5: Good wellbeing 178 no prior (32.5 percent), 370 prior (67.5 percent), total 548 (51.3 percent). Poor wellbeing 206 no prior (39.6 percent), 314 prior (60.4 percent), total 520 (48.7 percent).

Depression (P H Q dash 9): Unlikely depression 166 no prior (36.3 percent), 291 prior (63.7 percent), total 457 (42.8 percent). Likely depression 218 no prior (56.8 percent), 393 prior (57.5 percent), total 611 (57.2 percent).

Anxiety (G A D dash 7): Unlikely anxiety 222 no prior (32.9 percent), 453 prior (67.1 percent), total 675 (63.6 percent). Likely anxiety 159 no prior (41.7 percent), 228 prior (58.9 percent), total 387 (36.4 percent).

Readmission within 12 months: No 304 no prior (41.1 percent), 435 prior (58.9 percent), total 739 (67.1 percent). Yes 89 no prior (24.6 percent), 273 prior (75.4 percent), total 362 (32.9 percent).

Cluster: S M S 112 no prior (36.2 percent), 197 prior (63.8 percent), total 309 (28.1 percent). S M S plus P S 111 no prior (30.8 percent), 249 prior (69.2 percent), total 360 (32.7 percent). T A U 170 no prior (39.3 percent), 262 prior (60.6 percent), total 432 (39.2 percent).

Navigate back to Table 1..

### Table 2. Long description

Beginning at the top, the table lists characteristic categories: Study cluster, Inpatient satisfaction, Age, Gender, Ethnicity, Education, Relationship status, Employment, Housing, prior inpatient admission, B R S, W H O, Depression, Anxiety. Each category is subdivided by group (e.g., SMS, SMS plus PS, TAU for Study cluster; Very Satisfied, Satisfied, Not Satisfied for satisfaction). Columns from left to right are B, S E, Wald, d f, p value, O R, 95 percent C I (Upper Lower). Significant predictors include age greater than 40 years (B negative point five three two, p value point zero three four, O R point five eight seven, C I point three five eight to point nine six two), High School Diploma (B negative point eight six one, p value point zero two two, O R point four two three, C I point two zero three to point eight eight one), Post-Secondary Education (B negative point nine four zero, p value point zero one four, O R point three nine zero, C I point one eight four to point eight two eight), Unemployed (B point four one nine, p value point zero two five, O R one point five two zero, C I one point zero five four to two point one nine three), prior inpatient admission (B point six nine four, p value zero, O R two point zero zero two, C I one point four four eight to two point seven seven zero), and Other ethnicity (B negative point six five one, p value point zero five zero, O R point five two one, C I point two seven two to point nine nine nine). Other variables show non-significant p values. Odds ratios and confidence intervals are provided for each subgroup.

Navigate back to Table 2..
