# Supplementary material for: Predictors of psychiatric readmission within 12 months following discharge from inpatient units in Alberta, Canada
Source: Glob Ment Health (Camb). 2026 May 22;13:e129. doi: 10.1017/gmh.2026.10229 (PMC13373266; doi:10.1017/gmh.2026.10229)
Supplement: Elgendy et al. supplementary material [file S2054425126102295sup001.docx]

satisfactory

Thank you for completing this survey. The purpose of this survey is to receive feedback on your experience related to your current inpatient treatment. The information you provide will help us improve the service for you and others in the future.

This survey will take about 5 minutes to complete.Your participation is voluntary.Responses are anonymousThe information you provide will not be used to identify you – results will be reported as a group.We recommend that surveys be completed when connected to Wi-Fi.Completing the survey means that you agree to take part in the evaluation.

Please enter the mobile number (e.g., 780-123-1234)

that you would like to receive text message communication from the research team

Which Hospital are you currently or did you recently Edmonton


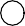

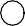

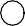


receive inpatient treatment? Calgary

Grande Prairie

Edmonton

Alberta Hospital Edmonton Royal Alexandra Hospital University of Alberta Hospital Grey Nuns Community Hospital Misericordia Community Hospital


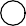

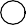

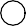

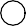

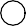


Calgary

Foothills Hospital


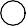

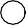

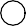

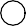


Peter Lougheed Center Rockyview Hospital South Heath Hospital

How long have you stayed in the mental health unit during the current hospital admission?

Less than one week One to two weeks


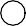

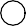

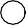

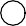

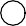


More than 2 weeks but less than 4 weeks 4 weeks to 8 weeks

More than 8 weeks

Did you feel welcome?

Was the hospital room you stayed in comfortable?

Did you receive consistent information about your treatment and care from staff?

Yes definitely Yes to some

extent

**Please choose an option that best describes your experience during this admission.**


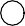

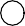

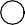

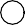

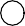

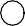


Neutral Not really Definitely not


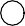

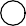

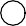

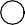

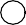

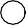

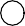

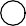

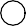


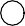

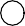

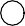

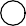

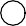
Did you feel the staff really listened to you?


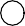

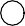

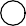

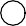
Did you feel the staff understood
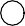
 your needs and concerns?


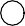

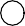

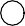

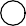

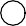
Did staff deal with your needs and concerns?


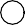

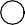

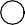

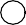

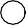
Did you feel the staff were genuine?


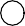

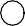

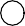

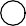

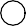
Did you find the staff knowledgeable?


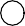

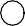

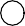

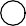

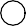
Did you feel that you were treated with dignity and respect?


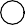

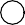

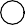

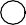

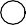
Were your preferences and values respected in your care (e.g., cultural, spiritual, gender, etc.)?

What was your involvement in the development of your Care Plan?

I was involved in the development of my Care Plan


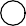

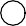

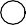

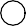


I was not involved in the development of my care plan but I reviewed and approved it

I was not involved in the development of my care plan and I have not reviewed my care plan I do not know if I have a Care Plan

How often have you reviewed your Care Plan with staff?

At least weekly Less than weekly Never


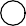

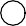

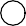


Did you feel you were given enough education/information about your condition or issue?

Yes definitely Yes to some

extent

**Please choose the best option which describes the Personalization and Effectiveness of the**

**Treatment received.**


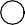

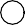


Neutral Not really Definitely not


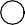

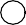

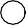


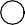

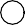

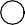

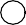

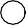
Were you given options or choices for your treatment and care?


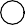

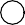

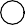

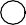

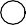

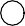

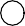

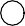

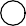

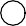
Was your treatment and care personalized to meet your needs?

Did you find the treatment and

care you received helpful?


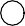

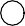

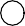

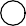

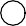
Did you feel your treatment and care helped reduce your symptoms and/or issues?


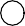
Did you feel you were involved enough in decisions about your treatment and care?

Who primarily supported you over the course of your Parent(s) inpatient care? Please select only one of the below Sibling(s)

options Partner(s)

Child/Children Friend(s) Counsellor/Therapist Social Worker Family Doctor Psychiatrist

AHS Service(s)

Non-AHS Service(s) Other

Please specify

Please specify

Please specify

Did staff include your family or someone close to you in your care as much as you wanted?

Yes definitely Yes to some

extent

**Please choose one option**

Neutral Not really Definitely not

Were you given enough time to talk about your condition or issue with staff?

Did staff tell you about the other services and supports available

to you during treatment if you needed them (e.g, physical health needs, child/family care, financial, housing, etc.)?

Did staff help you identify where to get support after you leave

the service (e.g., peer support, crisis management, etc.)?

Did your care appear to be well coordinated among all staff at this service?

Did you face any barriers when accessing this Yes

inpatient service? No

Please specify

Overall, how satisfied are you with the service you received?

Very satisfied Mostly satisfied Mixed

Mostly dissatisfied Very dissatisfied

We want to know the overall rating of your care and treatment. Using any number from 1 to 10, where 1 is the worst possible care and 10 is the best possible care, how would you rate your care and treatment at this program/service?

Worst possible Best possible

care care

*(Place a mark on the scale above)*

What did you like best about the service you received during your stay in the hospital?

What would you like to change about the service you received in the hospital?

Please add additional comments, suggestions, or questions here
