# Supplementary material for: Predictors of psychiatric readmission within 12 months following discharge from inpatient units in Alberta, Canada
Source: Glob Ment Health (Camb). 2026 May 22;13:e129. doi: 10.1017/gmh.2026.10229 (PMC13373266; doi:10.1017/gmh.2026.10229)
Supplement: Peer review history

# Reviewer report: Review: Predictors of psychiatric readmission within 12 months following discharge from inpatient units in Alberta, Canada — R0/PR2

**DOI:** 10.1017/gmh.2026.10229.pr2

**Authors:** Akira Sato

**Affiliations:** Independent Scholar, Japan

The authors demonstrated that several risk and protective factors for psychiatric readmission within 12 months after discharge, with particular interest in patient satisfaction with the index hospitalization. I was impressed by the authors' idea, which tried to include the lived experiences of inpatient stay from a readmission-prevention perspective. However, I was not certain whether this author’s view remained coherent throughout the manuscript.

Please consider rephrasing the title to reflect the contents of the manuscript. The authors used the word ‘correlates’ only twice in the title and in their aim in the introduction section; elsewhere, they often used other words, such as ‘predictors’. Also, strictly speaking, the word ‘rate’ describes the number of events per unit of time, whereas ‘risk’ or ‘proportion’ does not. I believe the authors should choose words that best describe their study. As Cambridge Prisms: Global Mental Health is intended for readers worldwide, please include the country where this study took place in the title.

In the abstract, please consider the overall structure. Namely, the authors' priority for patient satisfaction regarding readmission appears inconsistent across the subsections of the abstract. Moreover, the objectives of the abstract and the aim in the background section of the main text differ slightly but potentially significantly regarding patient satisfaction. The aim and objectives would shape the overall structure and logical flow of an article. Hence, the authors need to ensure that every section of the manuscript aligns with the aim and objectives. Additionally, include odds ratios with their confidence intervals for at least some of the predictors in the results section.

Please be consistent with the authors‘ intent throughout the background section. I believe readers of the manuscript would probably assume that examining the association between patient satisfaction and readmission is the authors’ primary focus until the readers reach the last two sentences in the background on page 4, where the emphasis on satisfaction suddenly disappears. If they intend to focus on patient satisfaction, they should explicitly include it in the aim. If the authors decide to keep the aim as it is, they must reconsider the overall structure of the background. In the latter case, please justify that this is a novel study, not merely a replication of previous studies. Also, the authors stated that “few studies differentiate between specific dimensions of patient satisfaction.” Please explain to what extent this study has successfully explored these specific dimensions. Furthermore, I could not find the gaps that the authors mentioned in the last sentence. Please kindly indicate what “these gaps” mean.

In the 2.1 Study Design and Data Collection section, please clarify the study design. It seems this study is part of a larger randomized trial, but it is not clearly stated. Also, state when the study participants took the survey during their hospitalization. I reckon the survey timing might affect the answers. For example, participants who used to be depressed or manic at hospitalization and answered the survey after stabilization would have answered differently if they had been asked the same question in the early phase of their inpatient stay. In the last sentence of this section, explain what the levels of mental health diagnosis are. The term only appears here. Additionally, all abbreviations should be spelled out on first use. Then, abbreviations are used afterwards throughout the manuscript. Please also justify why the authors developed their own patient satisfaction questionnaire rather than using already validated ones. In the 2.4 outcome measures section, be explicit about the authors' intent regarding the primary outcomes, as I mentioned in the background section.

Please include the number of excluded individuals and the reasons for their exclusion in the results section. If data are available, the authors could also explain whether there are any differences in characteristics between the included and excluded individuals. Also, please include the average number of hospital stays and the proportion of participants who were readmitted. For the latter, only the discussion section seems to display such numbers (i.e., 38.6% and 22.6% on page 12). As for Table 1, please state in the methods section that participants will be tabulated by prior inpatient admissions. Also, include the numbers of individuals with and without prior admissions in the first row of Table 1. Furthermore, consider relabeling the housing status in Table 1. I suppose that both participants who own a home and those who rent live alone, compared to the third row: living with family or friends. If so, I suggest noting that in the table.

In the discussion section, please clarify what priority the authors place on patient satisfaction in the context of previous studies. Throughout the manuscript, the authors‘ handling of patient satisfaction is unclear, obscuring the message the manuscript may convey to readers. Personally, I believe the authors should not hesitate to explicitly state that there was no association between patient satisfaction and readmission. The negative results can surely build on existing research, contributing, for example, to future systematic reviews. I suggest that the authors include a discussion of the possibility that the study design may have failed to detect the association statistically or to capture an appropriate dimension of patient satisfaction. Also, please add a discussion of patient satisfaction in relation to the literature that contradicts the authors’ results. The authors noted in the background that previous studies had mixed results regarding patient satisfaction.

Lastly, please consider the overall structure of the conclusion section and the impact statement, aligning them with the background, results, and discussion sections.

# Reviewer report: Review: Predictors of psychiatric readmission within 12 months following discharge from inpatient units in Alberta, Canada — R0/PR3

**DOI:** 10.1017/gmh.2026.10229.pr3

This manuscript presents an empirical study examining experiences and needs of users of psychiatric services based on a large dataset of respondents. The topic is highly relevant in the context of ongoing mental health service reforms and the growing emphasis on community-based care. The manuscript contributes valuable insights into the perspectives of service users and provides empirical evidence that may inform service planning and policy development. Overall, the study addresses an important research question and is based on a substantial dataset, which represents a notable strength.

Strengths of the Study

One of the key strengths of the manuscript is the large sample size (N=1070), which is relatively substantial for research focusing on users of psychiatric services. The inclusion of a large number of participants increases the statistical power of the analyses and allows for a more robust examination of associations between variables.

Another strength is the focus on service users’ perspectives. Much research in mental health services relies on administrative data or clinician perspectives, whereas this study directly captures the experiences and needs of individuals receiving psychiatric care. This approach contributes to a more patient-centered understanding of mental health services and aligns with contemporary principles of participatory and recovery-oriented care.

The study also addresses an important and policy-relevant topic. Understanding the experiences and needs of long-term users of psychiatric services is crucial for improving service organization, accessibility, and quality of care. The findings therefore have potential implications for mental health policy and the development of community-based support systems.

Furthermore, the manuscript appears to utilize multiple measures capturing different dimensions of service use and experiences, which allows for a more comprehensive analysis of the phenomena under investigation.

Limitations to Be Addressed

Despite these strengths and few limitations - such as the potential bias associated with self-reported data, limited generalization and represenatativeness - are adequately addressed in the limitations section, several limitations still should be acknowledged.

First, the study appears to employ a cross-sectional design, which limits the ability to draw causal conclusions. The associations observed between variables should therefore be interpreted cautiously, as the temporal direction of relationships cannot be determined. This is reflected in the name of article but should be also presented in the Limitation section and more reflected in the text, especially in the Conclusion section.

Second, the representativeness of the sample is still not entirely clear. Although the sample size is large, the manuscript does not fully clarify whether participants were recruited using a probability sampling approach or through convenience sampling.

Finally, the manuscript would benefit from a clearer description of the handling of missing data and the analytic sample used in the statistical model. At present, it is not entirely evident whether all participants provided complete responses for all variables included in the model.

Minor Revision Requests

Several minor issues related to reporting clarity should be addressed before publication.

First, Table 1 lacks a sufficiently detailed caption or description. The table appears to present counts and percentages of participant characteristics; however, the caption does not clearly specify what the percentages represent (percentages of the no prior / prior admissions subgroups).

Second, the manuscript should explicitly report the number of respondents included in the statistical model. While the total sample size is reported (N=1070), it is unclear whether all respondents completed all scales and variables used in the model. The current description of the study design and recruitment procedures does not clarify whether:

• all participants completed all measures,

• cases with missing data were excluded (listwise deletion),

• or missing data were handled using another method (e.g., imputation or FIML).

Providing this information would improve the transparency and reproducibility of the analysis.

Overall Evaluation

In summary, the manuscript addresses an important topic and is based on a large dataset of psychiatric service users. The study contributes meaningful insights into the experiences and needs of this population and has potential implications for mental health service development. However, several aspects of reporting require clarification, particularly regarding table descriptions and the analytic sample used in the statistical model. Addressing these minor issues would strengthen the clarity and transparency of the manuscript.

# Editor report: Recommendation: Predictors of psychiatric readmission within 12 months following discharge from inpatient units in Alberta, Canada — R0/PR4

**DOI:** 10.1017/gmh.2026.10229.pr4

**Affiliations:** Director, National Institute of Mental Health, Czech Republic

Overall, the reviewers assessed the manuscript positively; however, several points were raised that need to be addressed.

# Editor report: Decision: Predictors of psychiatric readmission within 12 months following discharge from inpatient units in Alberta, Canada — R0/PR5

**DOI:** 10.1017/gmh.2026.10229.pr5

**Affiliations:** Johns Hopkins University Bloomberg School of Public Health, United States

No accompanying comment.

# Reviewer report: Review: Predictors of psychiatric readmission within 12 months following discharge from inpatient units in Alberta, Canada — R1/PR7

**DOI:** 10.1017/gmh.2026.10229.pr7

Thanks for addressing all the methodological comments; it has significantly improved the quality of the manuscript and the interpretation of your findings. All reviewer comments have been addressed by the current revisions, and the manuscript can be accepted for publication.

# Reviewer report: Review: Predictors of psychiatric readmission within 12 months following discharge from inpatient units in Alberta, Canada — R1/PR8

**DOI:** 10.1017/gmh.2026.10229.pr8

**Authors:** Akira Sato

**Affiliations:** Independent Scholar, Japan

The authors have significantly improved their manuscript. Now it is much more coherent throughout and would provide insights into how mental health professionals can address the risk of readmission. For further improvement, allow me to point out some minor issues below:

Explain about predictor variables in the methods section. I believe that briefly mentioning the reasons for choosing the predictors and timing of data collection should suffice. Please cite references if necessary.

Consider including the number of excluded individuals as specified below. By ‘excluded individuals,’ I meant those excluded before randomization. I believe that the number helps readers interpret this study in their context. It typically appears in the second box from the top of the CONSORT flow diagram. Please refer to Figure 1 of the BMJ article (doi: https://doi.org/10.1136/bmj-2024-081123).

State the primary outcome at the very beginning of the results section, just after describing the baseline characteristics, rather than at the bottom of the section, as it is currently written in the manuscript.

Please include the average length of hospital stay in Table 1 or elsewhere. I regret mentioning it as the average “number” of hospital stays, which might have confused the authors. I meant the average number of days that patients spend in the hospital.

# Editor report: Recommendation: Predictors of psychiatric readmission within 12 months following discharge from inpatient units in Alberta, Canada — R1/PR9

**DOI:** 10.1017/gmh.2026.10229.pr9

**Affiliations:** Director, National Institute of Mental Health, Czech Republic

Accept the manuscript subject to minor revisions in line with the reviewer’s suggestions.

# Editor report: Decision: Predictors of psychiatric readmission within 12 months following discharge from inpatient units in Alberta, Canada — R1/PR10

**DOI:** 10.1017/gmh.2026.10229.pr10

**Affiliations:** Johns Hopkins University Bloomberg School of Public Health, United States

No accompanying comment.
